# Malaysian Society of Allergy and Immunology Consensus Statement on Sublingual Immunotherapy in Allergic Rhinitis

**DOI:** 10.3390/jcm12031151

**Published:** 2023-02-01

**Authors:** Baharudin Abdullah, Ruby Pawankar, Amir Hamzah Abdul Latiff, Kent Chee Keen Woo, Eike Wüstenberg, Mohamad Azizul Fitri Khalid, Yeoh Zhi Xiang, Salina Husain, Nurashikin Mohammad, Norasnieda Md Shukri

**Affiliations:** 1 Department of Otorhinolaryngology-Head and Neck Surgery, School of Medical Sciences, Universiti Sains Malaysia, Kubang Kerian 16150, Malaysia; 2 Division of Allergy, Department of Pediatrics, Nippon Medical School, Sendagi, Bunkyo-ku, Tokyo 113-8602, Japan; 3 Allergy & Immunology Centre, Pantai Hospital, Jalan Bukit Pantai, Kuala Lumpur 59100, Malaysia; 4 Allergy & Immunology, Gleneagles Hospital, Jalan Ampang, Kuala Lumpur 50450, Malaysia; 5 ALK-Abelló Arzneimittel GmbH, 22763 Hamburg, Germany; 6 Department of Otorhinolaryngology-Head and Neck Surgery, Dresden University, 01307 Dresden, Germany; 7 Department of Otorhinolaryngology-Head and Neck Surgery, Hospital Sultanah Bahiyah, Alor Setar 05460, Malaysia; 8 Department of Otorhinolaryngology-Head and Neck Surgery, Faculty of Medicine, Universiti Kebangsaan Malaysia, Kuala Lumpur 56000, Malaysia; 9 Department of Internal Medicine, School of Medical Sciences, Universiti Sains Malaysia, Kubang Kerian 16150, Malaysia

**Keywords:** allergic rhinitis, allergen immunotherapy, sublingual immunotherapy, subcutaneous immunotherapy, clinical practice recommendations

## Abstract

Allergic rhinitis (AR) is an IgE-mediated inflammatory disease of the upper airway. AR affects the patients’ quality of life, is a known risk factor for asthma and a socio-economic burden. Allergen-specific immunotherapy (AIT), comprising sublingual immunotherapy (SLIT) and subcutaneous immunotherapy (SCIT), involves administering increasing doses of the causative allergen to induce clinical and immunologic tolerance to the allergens. It is the only currently available treatment for AR that has been proven to induce disease-modifying effects (i.e., long-term remission of allergic symptoms or potential prevention of asthma and new sensitizations). Although AIT is conventionally recommended for patients who are non-responsive to symptom-relieving pharmacotherapy, it is presently recommended as a first-line treatment for patients with moderate to severe AR who prefer a treatment with the potential for long-term remission. In light of the relatively recent implementation of AIT in Malaysia, guidelines on its appropriate indication and application are important to attain optimal outcomes. This consensus statement was developed by an expert group formed by the Malaysian Society of Allergy and Immunology to provide evidence-based recommendations for the practice of AIT in Malaysia. Patient and product selection, choice of AIT, and strategy towards an effective treatment outcome in AIT are presented.

## 1. Introduction

Allergic rhinitis (AR) is one of the most prevalent allergic diseases, affecting around one-fifth of the general population, causing impaired quality of life of the patients and a marked socio-economic impact [1]. AR treatment consists of allergen avoidance, use of pharmacotherapy that provides symptomatic relief, and allergen-specific immunotherapy (AIT). Although allergen avoidance is one of the recommended treatments for AR, effective allergen avoidance may not be feasible. Pharmacotherapy is effective in controlling the symptoms of AR, but their effects are short-lasting and they do not alter the natural history of disease [2]. AIT is the only treatment that provides a disease-modifying effect in IgE-mediated diseases, such as AR and asthma, and its clinical benefits can persist for years even after discontinuing the treatment [3]. The clinical benefit of AIT has been shown to also improve patient quality-of-life parameters, including work productivity and sleep quality [4,5]. AIT has been recommended in patients who failed pharmacotherapy or were intolerant, and patients who prefer to avoid the prolonged use of pharmacotherapy [6,7].

AIT, given over a period of three years, has been shown to have persistent clinical benefits for several years after its discontinuation, as compared to conventional pharmacotherapy [2,8,9]. The disease-modifying effect of AIT is both antigen-specific and antigen-driven [9]. AIT engenders the production of IgG/IgG_4_, blocking antibodies which inhibit IgE-dependent activation of mast cells and basophils. This leads to the suppression of T_H_2 immunity due to a series of processes, including deletion of antigen-specific T cells, induction of antigen-specific regulatory T cells, or immune deviation, in favor of T_H_1 responses [9,10]. Long-term tolerance is further induced by the action of IL-10–producing regulatory B cells and ‘‘protective’’ antibodies [9].

Currently there are two main routes of AIT administration, subcutaneous immunotherapy (SCIT), and sublingual immunotherapy (SLIT). Both SCIT and SLIT are effective, with SLIT being particularly well-tolerated due to its lower risk of systemic allergic reactions, compared to SCIT [11,12,13]. SLIT can be applied as drop solution or sublingual tablet. Although SCIT was the more common form of AIT administration and had been the gold standard in AIT, it may require over 50 allergen injections throughout the treatment duration [14]. Lately, a growing number of patients are treated with SLIT products due to the well-established efficacy profile, lower rate of systemic allergic reactions and greater convenience for patients. Adherence to treatment is reportedly lower in SCIT, compared to SLIT, owing to the need for in-office administration of SCIT [15]. Apart from its effectiveness and better tolerance, SLIT offers self-administration at home, thus, providing a convenient option to patients [15]. A systematic review described statistically significant effects of both SCIT and SLIT, compared to the placebo across a number of outcome measures and the vast majority of subgroup analyses, with some results significantly favoring SLIT in children [16]. Economic modelling suggested that both SCIT and SLIT may be more cost-effective, as compared to symptomatic treatment after 6 years of treatment [16]. Another systematic review comparing the cost-effectiveness of SCIT and SLIT, highlighted that SLIT is a more cost-effective option in patients with > 40% compliance [17]. At present, SLIT has been registered in Malaysia and is available by prescription for the treatment of AR patients. With this choice, there is a necessity to produce a clear guide on the usage of SLIT to achieve optimum outcomes.

To establish a comprehensive and pragmatic approach on the use of SLIT in the treatment of AR patients, a consensus statement was commissioned by the Malaysian Society of Allergy and Immunology (MSAI) and developed with a panel of experts.

## 2. Methodology

A virtual meeting of a panel of ten experts was held in February 2022 to discuss and provide a consensus. The panel consisted of six otorhinolaryngologists, two allergists, one respiratory physician, and one pediatrician. All the experts involved were recognized by their fraternities as experts in the field of allergy and have sufficient experience in treating patients with allergic rhinitis. The expert panelists involved had background records of research publications, presentation and involvement in allergy meetings/conferences, and working experience in treating allergic rhinitis for at least 3 years. To support the consensus and recommendations, the panel of experts subsequently reviewed evidence from the available literature on AIT, which included both SLIT and SCIT, to provide a comprehensive overview of the treatment algorithm. Medical literature search was performed on the PubMed database. The search strategies used the following keywords either separately or in combination: allergen immunotherapy, allergic rhinitis, sublingual immunotherapy, subcutaneous immunotherapy, duration, patient selection, symptoms, affordability, adverse effects, and asthma. Studies and abstracts in the English language published up to February 2022 were reviewed. Relevant meta-analysis, randomized controlled trials, and observational studies were included. Review articles, editorials, case series, and case reports were excluded. The consensus and recommendations were based on the literature review together with expert opinion. The consensus statements consisted of definitions, requirements, and recommendations that met a minimum requirement of 70% agreement among the panelists.

## 3. Consensus Recommendations

### 3.1. Selection of Patients

AIT is only indicated when there is evidence of an immunoglobulin E (IgE)-mediated disease that correlates with the AR symptoms [3]. The European Academy of Allergy and Clinical Immunology (EAACI) guidelines recommended the usage of a positive diagnostic test (allergen-specific IgE or skin prick test), interpreted according to the patient’s clinical history of AR symptoms, as a requirement for the initiation of AIT [3]. The guidelines also specify that the patient must exhibit refractory symptoms despite the use of pharmacotherapy and patient preference towards AIT should also be taken into consideration [3]. In light of these recommendations, AIT should be offered to patients with uncontrolled AR despite the use of pharmacotherapy and proven IgE sensitization by either serum IgE or skin prick test [3].

Several guidelines and international consensus documents recommend the use of AIT in the context of pharmacotherapy failure or intolerance [1,3,7,18]. However, based on the severity of AR (moderate to severe) and presence of comorbidity, patients can be offered AIT as a first-line of treatment for AR at initial presentation if this is a preferred option (depending on availability and affordability of product). This recommendation is based on the disease-modifying and long-lasting effects of AIT and the potential to prevent co-morbid conditions [8]. Furthermore, long-term pharmacotherapy may be associated with its own set of adverse effects [7].

The characteristic pattern of the patients’ symptoms may be associated with specific allergens, e.g., symptoms worsening at a specific time at night or morning may indicate a house dust mite allergy, in contrast to pollen allergy, where symptoms occur throughout the day [19]. A systematic review and meta-analysis performed with AR patients has reported a significant reduction in Rhinitis Total Symptom Score with house dust mite SLIT tablet [20]. In addition, it is interesting to note that a study performed in a single Malaysian center has reported that ethnicity may also influence the symptom severity of AR. The Malaysian study found that symptoms of AR were most severe among patients of Malay ethnicity and at least as severe among those of Chinese ethnicity [21]. However, while symptom severity may vary depending on ethnicity, the AIT effect is not anticipated to be similarly influenced and is applicable to all irrespective of racial or ethnic origin.

When selecting patients for AIT, patient preference and affordability of the AIT product should be taken into account [1,3]. Young patients should be able to comprehend the application of SLIT administration (i.e., placed beneath the tongue for a period of time and not to be immediately swallowed). It is also important to note that the repeated injections of SCIT may not be tolerated well by young children [22,23]. In addition, pregnant women should not start AIT during their pregnancy. However, the continuation of well-tolerated AIT during pregnancy is considered to be safe, although it should be performed with caution [24,25,26]. A prospective study on the safety of SLIT in pregnant women reported a lower incidence of complications with SLIT, as compared to the two control groups (budesonide 400 µg twice daily or rescue salbutamol inhalation) and the general population [24].

Several studies supported the use of AIT in treating patients with local AR [27]. If this is being considered, nasal allergen provocation testing is recommended for confirmation of local AR prior to commencing treatment. If there are concerns of risk of asthma exacerbation or bronchial hyperresponsiveness, especially in patients with long standing and severe AR, a pulmonary function test is helpful prior to the initiation of therapy. Nonetheless, this is not an absolute requirement for all patients.

The administration of SCIT is not indicated in severe asthmatic patients [3]. In a prospective trial of house dust mite SCIT in patients with asthma, asthma exacerbation occurred in 73.3% of patients with FEV1 (forced expiratory volume in 1 s) < 80%, as compared to only 12.6% of subjects with FEV1 > 80% (*p* < 0.0001) [28]. Therefore, in patients with severe asthma (FEV1 < 80%), clinicians must evaluate prudently and initiate AIT with caution.

**Figure 1:**
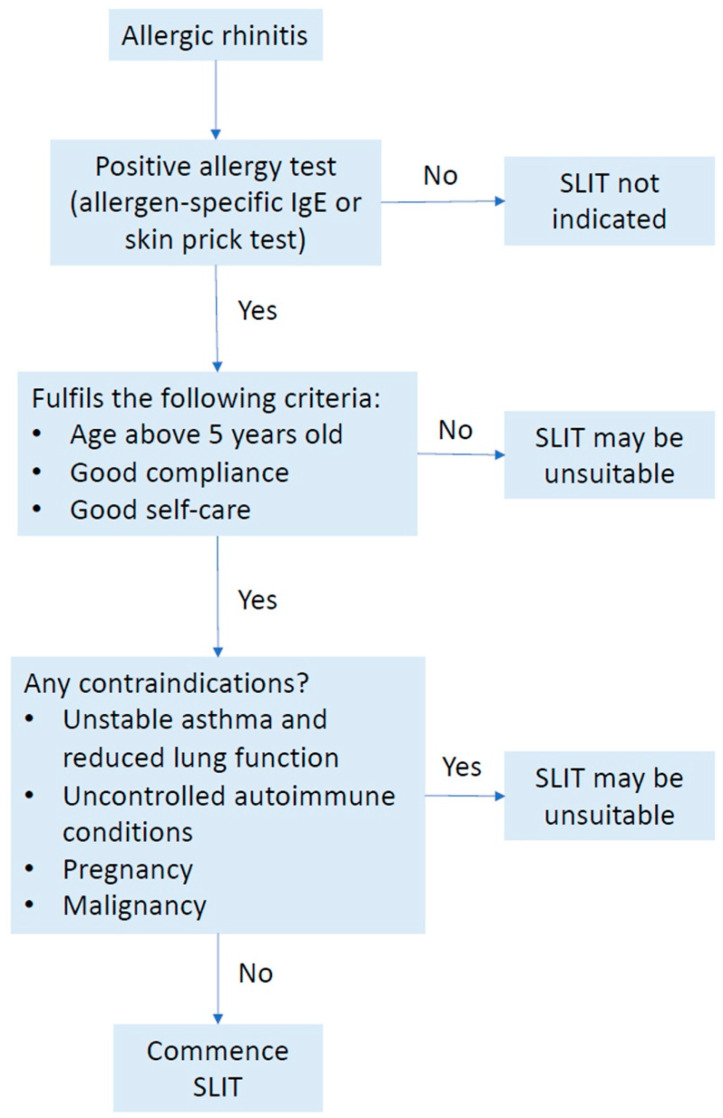
Algorithm to select suitable allergic rhinitis patients for sublingual immunotherapy. IgE, immunoglobulin E; SLIT, sublingual immunotherapy.

In order to clearly elucidate the characteristics of AR patients who may be offered SLIT (or cases when SLIT is contraindicated), we propose an algorithm, as shown in Figure 1.

#### Recommendation 1

1. AIT is offered to patients experiencing uncontrolled AR, despite the optimal use of pharmacologic treatment with proven IgE sensitization by serum IgE and/or skin prick test.
2. AIT can be offered as an option for first-line treatment of moderate to severe AR at initial presentation, if this is preferred by patients.
3. Patient’s preference and affordability of treatment must be given due consideration.

### 3.2. Selection of Products

Although several clinical trials and meta-analyses have shown evidence to support the efficacy and safety of AIT, the trials are highly heterogeneous, particularly in the type and quality of allergen product used, treatment schedules, and the target populations [29]. This heterogeneity affects the interpretation of the overall conclusions made in regard to each of the individual product. More reliable evidence derived from large clinical trials for a specific product and a commonly used dose schedule is imperative [29,30].

Consequently, the use of registered products is crucial and recommended in product selection. Certain SLIT products may be unregistered in many countries due to lack of available clinical data required for registration [31]. This can also mean that these products do not have sufficient evidence of their clinical efficacy. Some products (e.g., peanut or cat dander SLIT) are supported only by small-scale clinical studies (only tens of patients in each arm), although others (e.g., grass, ragweed or house dust mite SLIT) are supported by high quality evidence [32,33,34,35,36,37,38].

The World Allergy Organization and the EAACI guidelines both recommend product-specific evaluation; only products with documented efficacy should be used [3,39]. Therefore, recommendations to use AIT should be product-specific, instead of a broad SLIT/SCIT recommendation. Clinicians should opt to use registered products with adequate supporting clinical data in reasonably sized, double-blind, placebo-controlled studies [40]. The use of unregistered products must be limited to only when no registered products are available [41]. In clinical practice, it is obligatory that patients consent to treatment, but consent is especially critical when opting to use unregistered products [42]. In order to enable better access to AIT, patients should be enrolled in named patient programs if such programs are available.

#### Recommendation 2

4. Recommendations to use AIT products should be product-specific.
5. Use only registered products backed by adequate supporting clinical data from double-blind, placebo-controlled studies.
6. Written informed consent is essential prior to the initiation of AIT.

### 3.3. Selection of AIT (SLIT vs. SCIT)

Whereas SCIT is always administered in a clinical setting by healthcare professionals, only the first dose of SLIT is administered in-office; subsequent doses of SLIT are self-administered by patients or caregivers at home [3]. Unlike SLIT, SCIT has not been registered in Malaysia, and its use in the country is currently restricted to named patient programs.

SLIT and SCIT should be administered for a minimum period of three years to achieve optimal efficacy and sustained long-term benefit [8,43,44]. In a study randomizing AR patients to either one, two, or three years of house dust mite SLIT, the three-year course was proven to be more efficacious than the one- or two-year courses [43]. A five-year prospective study involving children with dust mite respiratory allergy reported that three years of SCIT produced a significant improvement, although a five-year course produced further improvement in symptoms [44]. In a prospective open controlled study following patients with mite allergy for 15 years, three years of SLIT conferred a clinical benefit persisting for seven years, whereas four and five years of SLIT both produced a clinical benefit persisting for eight years [8]. Furthermore, the symptom relief can be seen as early as 12 weeks after initiation of treatment [23]. It should be noted that the safety profile of SLIT is more tolerable, compared to SCIT when it comes to systemic allergic reactions [11,12,13]. Anaphylaxis during SLIT is infrequent, compared to SCIT, though it cannot be ruled out [13,45,46]. Local allergic reactions in SLIT, due to the mode of action, are to be expected but are normally mild to moderate in intensity. They typically diminish within a few weeks with continued use as a first sign of tolerance induction and may be reduced by taking oral antihistamine prior to or after administration [3,47].

In choosing the type of AIT for AR patients, patient preference plays an important role. A survey carried out among AR patients highlighted factors such as the absence of risk of systemic allergic reaction and a non-invasive route of administration as the most important attributes when making their choice for AIT [48]. Prior to commencing AIT, patients should be informed of the benefits of AIT, the application of AIT, its onset of action, efficacy, duration of treatment, cost, most likely adverse effects and risks, frequency of clinic visits required, and any possible alternatives to AIT in order to facilitate making a shared decision between clinician and patient to initiate AIT [3,49]. Sufficient patient education and healthy communication between clinician and patient helps in selecting the right therapy and ensure treatment compliance [3].

In view of SLIT being self-administered, patients on SLIT and their caregivers must be cautioned of adverse events, with clear information and instructions given regarding the steps to take if any adverse events were to occur while at home [3,49]. Table 1 summarizes the main differences between SLIT and SCIT.

#### Recommendation 3

7. Patient preference plays an important role in the choice of AIT after weighing the risk and benefit.
8. Prior to commencing AIT, crucial information must be communicated to the patients.

### 3.4. Strategy towards an Effective Treatment Outcome in AIT

For both SLIT and SCIT, patients must be informed of the treatment concept, the occurrence, and nature of adverse events, as well the onset of efficacy after 3–4 months of treatment (although this may vary amongst individual patients and used products). Clinicians must also emphasize to patients the importance of maintaining treatment for at least a minimum of three years [8,43,44]. Patients and caregivers must be adequately well-informed in order to manage expectations and allay fears [3,49]. Good communications between clinician and patient are critical to improve compliance and treatment outcome [3]. Table 2 shows a checklist in prescribing AIT to guide clinicians in their practice.

We recommend that the first SLIT dose is taken without premedication in order to observe for any adverse reactions in-office. After monitoring reactions without premedication at the first dose, premedication (oral antihistamines or systemic corticosteroids) may then be provided at subsequent dosing; otherwise, patients may continue without premedication if not required. Premedication can be prescribed for 3–4 weeks for patients who require it, although the duration of prescription may vary according to individual patients. Clinicians must reassess the need for premedication at subsequent visits. Patients may also be given rescue medications for their symptoms. Furthermore, patients must be informed that premedication does not interfere with AIT efficacy, as some patients have the impression that premedication ‘neutralizes’ the effect of AIT. Some patients may refuse premedication based on the assumption that they have ‘moved on’ to another type of medication, i.e., AIT, and, therefore, no longer require other medications.

Supervision of the first intake of SLIT is key in order to ensure correct administration and secure compliance. Patients need to be instructed where exactly to place the SLIT tablet/drops under the tongue. After the first SLIT tablet intake, a 30 min waiting time in the office is recommended. Adverse effects (e.g., local allergic reactions) with SLIT are common, although a multi-center, randomized, placebo-controlled study of grass SLIT reported that adverse effects usually resolve after 1–2 weeks of treatment (again, this may vary between individual patients) [35]. Discontinuation due to local allergic reactions also typically occurred within the first several weeks of treatment [35]. Patients on SLIT who administer doses at home should have access to telecommunication assistance and the opportunity to be seen promptly should the need arise.

Treatment outcomes (e.g., change in symptoms) may become apparent starting from a few weeks to months after initiating SLIT, although this may vary according to individual patients. For seasonal allergens, starting treatment 3–4 months in advance may be required for efficacy; grass SLIT, for example, are provided to patients starting 16 weeks prior to each grass pollen season [50,51]. Clinicians are recommended to schedule a follow-up 3–4 weeks after initiating SLIT to review the progress of treatment and to ensure compliance. After a few weeks or months, most initial local allergic reactions are expected to have subsided, which brings a positive point of discussion with patients during follow-up appointments. Symptomatic reduction and improvement is sensibly expected within the first year of therapy [52]. Failure of response within this time frame warrants reevaluation of diagnosis, therapy, dosing, and adherence. Regular monitoring is essential every 3–6 months to substantiate the benefit of SLIT or to consider stopping treatment when indicated.

Depending on the severity, the local adverse effects can be graded from one to three, while the systemic effects can be graded from one to five [53,54]. The specific grading criteria of the potential adverse effects of SLIT are summarized in Table 3. In the eventuality of oropharyngeal infections, oral lesions or inflammations, and dental extraction or oral surgery, SLIT should be withheld temporarily to allow the healing of the oral cavity [52]. Similar counsel is specified for acute gastroenteritis or exacerbations of asthma. A significant clinical concern with the use of SLIT is the development of eosinophilic esophagitis, which is the most common reason for treatment discontinuation in trials [55]. Eosinophilic esophagitis is considered a contraindication for SLIT and the discontinuation of immunotherapy is necessary when eosinophilic esophagitis is suspected [56].

#### Recommendation 4

9. The first SLIT dose should be taken in-office, and patients must be supervised for at least 30 min after the first dose. Patients on SCIT, on the other hand, must be observed after every dose.
10. The first AIT dose may be taken without premedication to observe for adverse reactions.
11. Prescribe premedication and rescue medication for needful patients.
12. Follow-up of SLIT patients after 3–4 weeks to discuss progress of treatment and to ensure compliance with further regular follow up according to individual patients.
13. Patient education on AIT is essential.

### 3.5. Unmet Needs and Gaps in Research/Knowledge

There is a need for multi-center randomized controlled trials in Malaysia for not-yet-registered specific SLIT/SCIT products, focusing on treatment efficacy and patients’ quality of life. These trials would be beneficial to strengthen the level of evidence available for AIT products. Robust data would permit these products to be registered in the country, encourage government funding for AIT, and convince insurance policymakers to extend coverage for AIT. All of these would improve the patients access to immunotherapy.

Many patients, as well as healthcare professionals, are still unaware of AIT and its potential benefits, which contributes to the low rate of AIT acceptance among Malaysian patients. In addition to the use of local guidelines [57], training courses for the health care professionals on the application of AIT are needed to facilitate the dissemination of appropriate information to patients.

AIT is often not offered as an option to AR patients in Malaysia. In the local government healthcare setting, AIT is not subsidized, and in the private setting, only 20% of patients pay out of their pocket for medications (the other 80% are covered by insurance). However, it should be noted that patients attending public hospitals, as well as patients with medical insurance from private facilities, have frequently expressed interest in AIT when it is offered as an option, despite this constraint.

## 4. Conclusions

By treating the allergic disease directly at the level of the immune system, AIT provides a valuable alternative to pharmacotherapy in the management of AR. It is indicated in patients with proven IgE-mediated disease that correlates with their AR symptoms. AIT may be offered to patients regardless of the severity of disease and also as a first-line treatment, based on the strength of evidence of its disease-modifying effect and potential to prevent co-morbid asthma and new sensitizations. When considering initiating AIT, patients’ preference and product affordability must be taken into consideration. Consequently, patients must be informed regarding the treatment concept and the benefits of AIT. It is recommended to use only registered products with proven clinical efficacy. SCIT or SLIT should be administered for a minimum period of three years. Patients must be supervised after each dose of SCIT, and at the first dose of SLIT. Adverse reactions can be managed with premedication, such as oral antihistamines or systemic corticosteroids, and patients may also be provided with rescue medication when needed. In order to strengthen the level of evidence for not-yet-registered products available for the use of AIT in Malaysian patients, clinical trials are needed to obtain robust data. Finally, we consider these recommendations are pivotal to galvanize the access and administration of AIT among Malaysian patients.

## Figures and Tables

**Table 1:** Overview of the differences between SLIT and SCIT.

SLIT Tablets/Drops	SCIT Injections
No up-dosing required (depending on product)	Up-dosing required
Mostly local allergic reactions, which subside with continued use, though systemic reactions may also appear	More systemic and local adverse reactions
Less time consuming for doctor and patients	More time consuming
No office visits required (except the first administration in-office)	Office visits required followed by at least 30 minutes’ waiting time after each injection
Administered daily by the patient	Administered monthly by the doctor (up-dosing weekly)
Non-invasive approach	Invasive approach

Abbreviations: SLIT, sublingual immunotherapy; SCIT, subcutaneous immunotherapy.

**Table 2:** Checklist guide for healthcare professionals when prescribing allergen immunotherapy.

Stable asthma
No contraindications, according to product information leaflet
Confirmed sensitivity by skin prick or serum IgE test
First dose intake of SLIT performed in-office

Abbreviations: IgE, immunoglobulin E; SLIT, sublingual immunotherapy.

**Table 3:** Mild-to-severe grading for potential systemic and local adverse effects with SLIT.

Systemic Reactions	Grade 1	Grade 2	Grade 3	Grade 4	Grade 5
	Involvement of no more than 1 organ/system as follows: Cutaneous Skin reaction or changes apart from the administration site And/or Tingling or itching of the lips or mouth Or Angioedema without laryngeal involvement OR Upper respiratory Nasal manifestation of rhinorrhea, pruritus, congestion And/or Tickling throat sensation or itchiness And/or Cough without bronchospasm OR Erythema, tearing, or pruritus of conjunctiva OR Nausea or metallic taste	Involvement of 2 or more organs/systems as stated in grade 1	Evidence of mild bronchospasm that is responsive to treatment, such as shortness of breath, cough, wheezing AND/OR Gastrointestinal symptoms, e.g. cramps, vomiting, diarrhea Others Presence of grade 1 symptoms/signs	Lower airway manifestation, e.g., severe bronchospasm, unresponsive or deteriorating despite treatment AND/OR Upper airway manifestation such as laryngeal narrowing Presence of grades 1 or 3 symptoms/signs	Respiratory failure AND/OR Hypotension And/or Loss of consciousness Presence of grades 1, 3 or 4 symptoms/signs
Local reactions	Grade 1	Grade 2	Grade 3		
Clinical features of dysgeusia, oral/ear pruritus, swollen lips/tongue, mucosal/pharyngeal oedema, glossodynia, mouth/tongue ulcer, throat irritation, abdominal pain, vomiting, nausea, diarrhea	Not burdensome AND Treatment not required AND SLIT to proceed	Burdensome OR Treatment required AND SLIT to proceed	Burdensome OR Treatment required AND SLIT suspended		

Abbreviations: SLIT, sublingual immunotherapy.

## Data Availability

This study did not generate any datasets.
